# Supplementary material for: TRIM26 Facilitates HSV-2 Infection by Downregulating Antiviral Responses through the IRF3 Pathway
Source: Viruses. 2021 Jan 6;13(1):70. doi: 10.3390/v13010070 (PMC7825454; doi:10.3390/v13010070)
Supplement: Supplementary file 1 [file viruses-13-00070-s001.pdf]

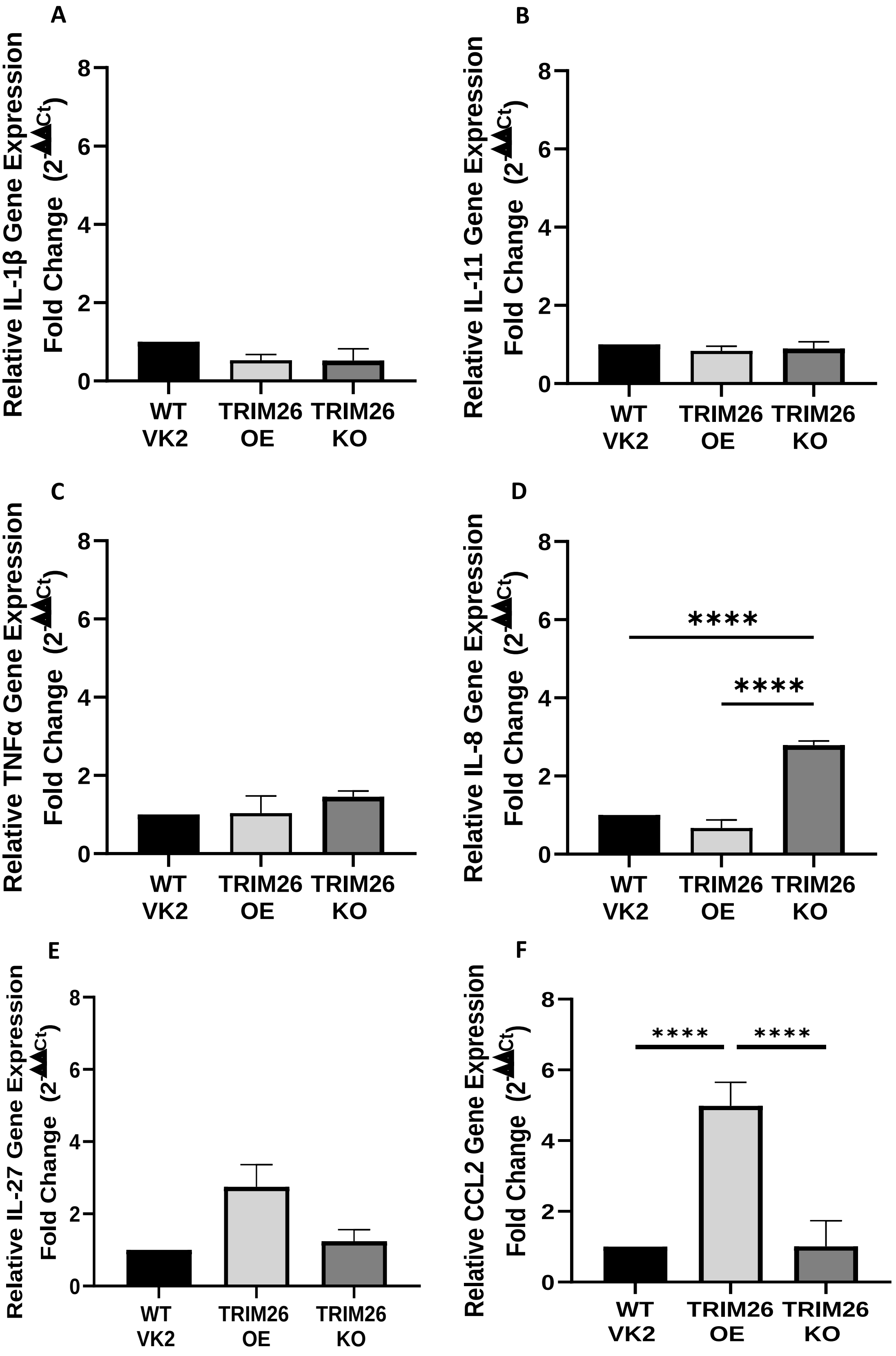

Supplementary Figure 1: Cytokines and chemokines produced by WT VK2, TRIM26 OE and TRIM26 KO. RNA from all three cell lines: WT VK2, TRIM26 OE and TRIM26 KO were subjected to qPCR using specific primers for different cytokines and chemokines (A) IL-1β, (B) IL-11, (C) TNF-α, (D) IL-8, (E) IL-27 and (F) CCL2. Fold change relative to VK2 WT are shown. Representative data is shown (n=3). ****$p<0.0001$.

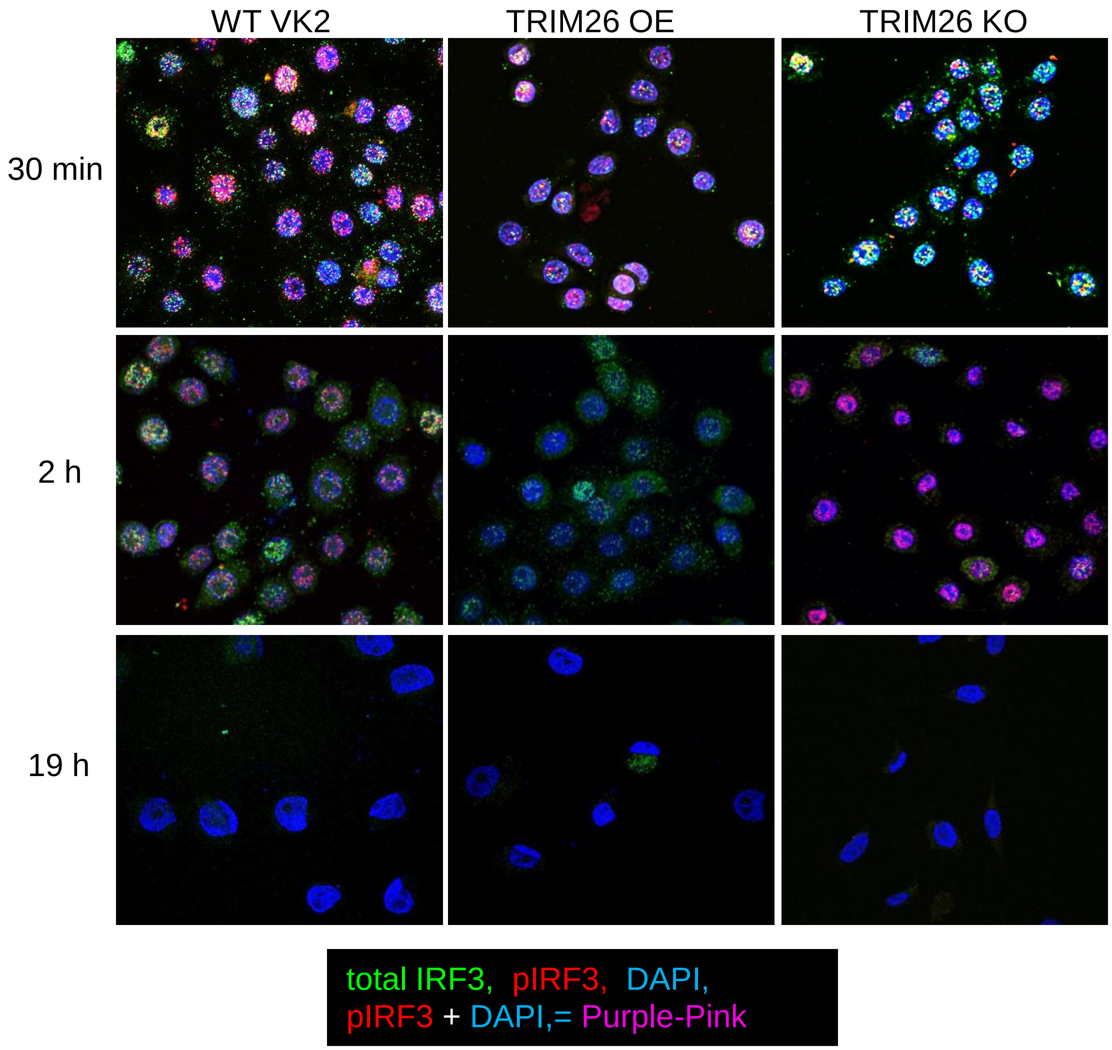

Supplementary Figure 2: IRF3 is activated in early hours of infection. All three cell lines WT VK2, TRIM26 OE and TRIM26 KO were infected with HSV-2 strain 333 (MOI = 1) and fixed after different time points. Fixed cells were stained for total IRF3 (green) and phosphorylated IRF3 (red) and nuclei (blue). Images were captured with confocal microscope at a magnification of X630. Representative images are shown. All three cell lines show an early activation of IRF3 (formation of phosphorylated IRF3) within 30 minutes of HSV-2 infection. After 1-2 hours TRIM26 OE showed loss of phosphorylated IRF3 and its accumulation in the nucleus, while WT VK2 and TRIM26 KO still show continued accumulation of phosphorylated IRF3 in the nucleus. Later time point (19 h) staining showed very little total IRF3 and no activated or phosphorylated IRF3 in all three cell lines.

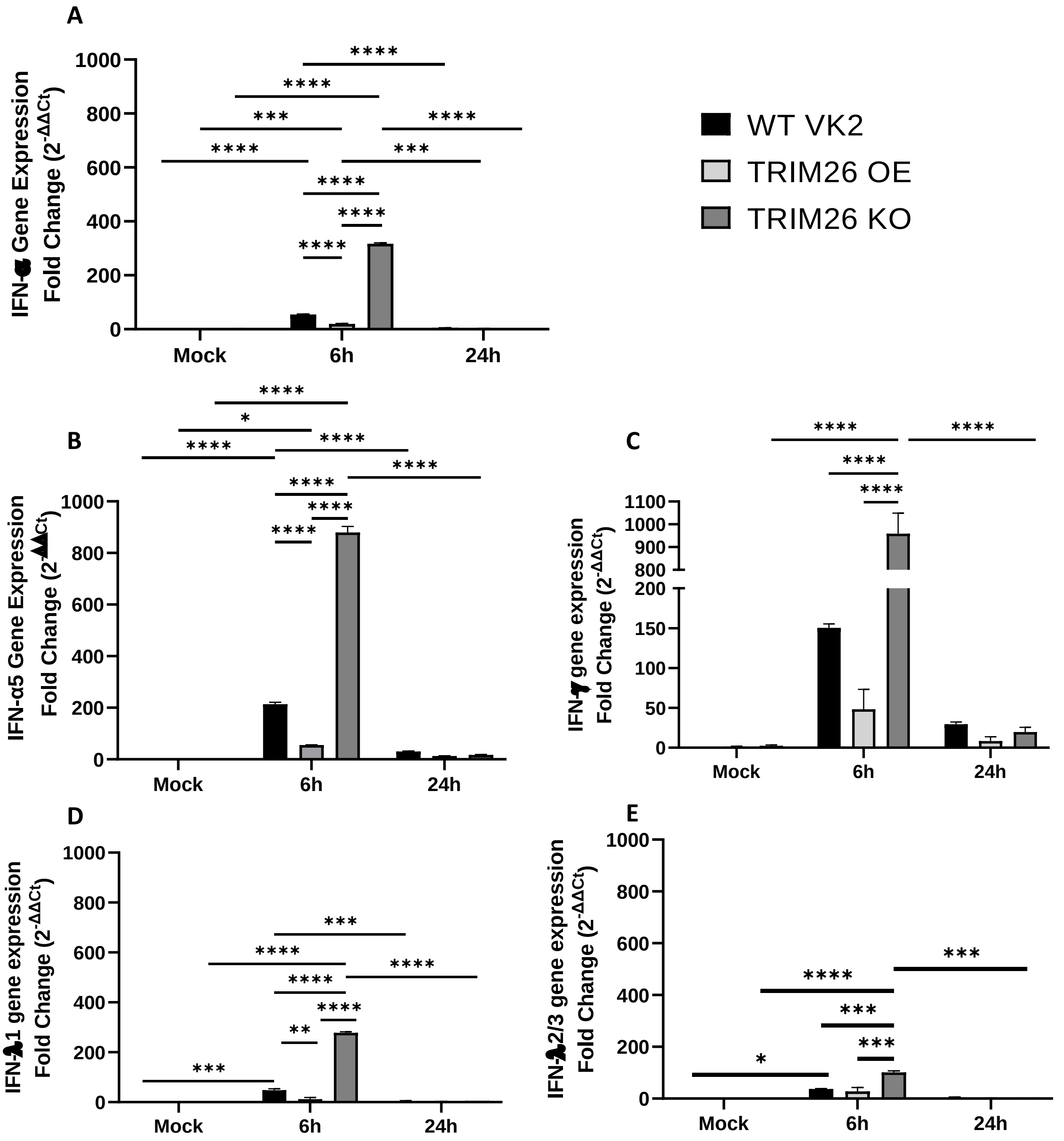

Supplementary Figure 3: HSV-2 infection upregulates IFN-a, IFNa5, IFN-γ and IFN-λ1 and IFN-λ2/3 in vaginal epithelial cells. All three cell lines WT VK2, TRIM26 OE and TRIM26 KO were infected with HSV-2 strain 333 with an MOI of 1 and RNA was extracted at different time points and subjected to qPCR using primers of IFN-α (A), IFNα5 (B), IFN-γ (C), IFN-λ1 (D) and IFN-λ2/3 (E). Fold change relative to uninfected (mock) controls are shown. Data represent mean ± SEM (n = 3). Representative data is shown (n=3). **p=0.002, ***p=0.001, ****p<0.0001. Figures show either analysis of significance between different cell lines with same time points or between different time points with same cell lines.
